# Supplementary material for: COmmunities Facilitating incREasing Smoke-free Homes (CO-FRESH): co-developing a toolkit with local stakeholders in Indonesia and Malaysia
Source: BMJ Glob Health. 2025 Oct 5;10(10):e018102. doi: 10.1136/bmjgh-2024-018102 (PMC12506144; doi:10.1136/bmjgh-2024-018102)
Supplement: online supplemental file 5 [file bmjgh-10-10-s005.pdf]

Supplementary file 5

Duration of measurement, median, minimum and maximum $PM_{2.5}$ values measured in each home

Home	Duration of sampling (days)	Mean $PM_{2.5}$ ($\mu g/m^3$)	Median $PM_{2.5}$ ($\mu g/m^3$)	Minimum $PM_{2.5}$ ($\mu g/m^3$)	Maximum $PM_{2.5}$ ($\mu g/m^3$)
**Indonesia**					
1	8	57	32	3.6	1036
2	8	89	33	3.0	1737
3	7	55	33	3.4	943
4	7	62	35	4.4	1351
5	7	42	35	2.4	290
6	7	43	29	1.9	783
7	7	37	28	2.2	508
8	7	64	44	7.3	576
9	7	53	38	7.0	936
10	7	46	31	6.6	712
11	8	51	35	1.7	836
12	8	72	43	0.8	1212
**Malaysia**					
1	7	32	22	8.8	1098
2	6	39	27	10.5	1071
3	7	29	23	9.0	314
4	7	18	14	5.8	305
5	7	32	21	8.2	252
6	6	16	14	6.0	316
7	7	34	26	7.9	469
8	7	43	31	20.5	344
9	7	36	30	16.4	560
10	7	28	23	8.9	168
